# A Content Analysis of Gambling Operators’ Twitter Accounts at the Start of the English Premier League Football Season

**DOI:** 10.1007/s10899-019-09879-4

**Published:** 2019-08-03

**Authors:** Elizabeth A. Killick, Mark D. Griffiths

**Affiliations:** International Gaming Research Unit, Psychology Department, Nottingham Trent University, Nottingham, UK

**Keywords:** Gambling, Social media, Marketing, Advertising, Sports betting, Online sports betting

## Abstract

The use of social media is now an established strategy to engage and maintain customer loyalty. The purpose of the present study was to examine the Twitter accounts of ten of the largest online sports betting operators in the UK to determine what marketing strategies were employed. More specifically, this study analyzed 3375 tweets posted by the operators during the opening weekend of the 2018–2019 English Premier League football season using a content analysis methodology. The results demonstrated that multiple strategies, including hashtags, were used to link gambling operator tweets with major sporting events, and the use of numerous promotional campaigns. Notably, over 90% of the tweets contained no responsible gambling information. The quantity and content of social media posts underline the need for a review of the current advertising regulations in the UK. Further research should examine how exposure to sports betting social media marketing influences gambling behavior.

## Introduction

Participation in online sports betting in the UK is steadily increasing, and due to changing consumer behaviors and technological advancements, this trend is expected to continue (Gambling Commission 2018a). Social media use has markedly grown over the past decade, and this is expected to continue (Duggan et al. 2015). One of the most popular social media sites is Twitter, a microblogging platform in which individuals, organizations, and commercial operators share news, information, personal opinion, seek information (Java et al. 2007) and/or meet emotional needs (e.g., Stieglitz and Dang-Xuan 2013). Twitter has over 326 million global monthly active users (Twitter 2019), with approximately 13 million users based in the UK (Aslam 2019) where the present study was carried out. Of these, there are approximately 40% female users and 60% male users. Twitter allows individuals to post short text messages (i.e., ‘tweets’) to people who have chosen to ‘follow’ the sender. Followers can actively engage and ‘retweet’ (i.e., share the post with followers), ‘like’, and/or ‘reply’ to posts. Hashtags denote a specific topic for users to participate in or follow the online conversation. More recently (and because of the large potential audience), social media has become an increasingly used marketing platform which many businesses utilize to connect with (and directly market to) current and prospective customers.

Twitter has gained a large following among sports fans (Price et al. 2013). For example, in the 2016 UEFA (Union of European Football Associations) European Championship, more than 14 million tweets were sent in one night when Portugal beat France to win the competition, resulting in 109 million tweets in total relating to the #EURO2016 (Collin 2016). Twitter is also a popular social media platform used by gambling operators (Gainsbury et al. 2015a). Research has found that 1 in 20 Twitter users in the UK follows at least one account dedicated to producing content promoting gambling (Miller et al. 2016). Additionally, it appears that the number of social media followers of gambling operators is increasing. For example  in 2013, *Paddy Power*, the Irish gambling operator, had over 1.7 million Facebook fans and Twitter followers, half of which were existing customers (Lauchlan 2013). This has now increased to over 2.1 million followers across the two platforms at the time of writing in March 2019.

Twitter requires that its users are over the age of 13 years. However, there are at least 200,000 children under the age of 12 years who are active monthly users on Twitter (Titcomb 2018). Additionally, statistics reported from the UK Gambling Commission show that 12% of 11 to 16-year-olds follow one or more gambling operator on social media (i.e., Facebook, Instagram and YouTube) (Gambling Commission 2018b). Of this group, 34% had spent their own money on gambling (in the last week) and were three times more likely to have done so compared to those who did not follow a gambling operator on social media. It is important to note that this research did not include statistics relating to Twitter use by youth. However, in a separate report, Ofcom (2017) reported that 19% of all children aged 12–15 years had accessed Twitter at some point in 2017.

Given the popularity of social media amongst young people, it is conceivable that children and adolescents will be exposed to the online gambling market. Previous research has found that 70% of children in the UK recall seeing a gambling advert on social media (Gambling Commission 2018b). Similarly, an Australian study of basketball fans aged 11–16 years found that 55% were able to recall seeing a gambling advert on social media (Thomas et al. 2018). These findings have resulted in concerns relating to the (i) exposure of gambling advertising via social media to young people, (ii) volume of gambling advertising, and (iii) normalization of gambling (Gambling Commission 2018b).

In relation to gambling advertising regulations on Twitter, the advertising of gambling products within the UK is permitted providing that the gambling operator holds a license from the UK Gambling Commission, the regulatory body formed after the 2005 Gambling Act. Over the years there have been a number of reviews on gambling advertising research (e.g. Binde 2014; Griffiths 2005; Newall et al. 2019; Parke et al. 2015) and it has been noted that gambling marketing is selectively targeted at sports fans, who may find it hard to avoid (Newall et al. 2019). Another review of gambling advertising noted that there is concern that social media marketing may contribute to an increase in the number of people gambling and possible gambling-related harm (Parke et al. 2015).

The Gambling Commission (2019) reported that Facebook was the most popular social media platform followed by online gamblers (19%), followed by YouTube (9%) and Twitter (8%). Research using Twitter data is relatively novel and there are no established or consistent methods across the few studies that are available. However, a few studies have explored sports betting advertising on social media, including Twitter (e.g., Gainsbury 2015b; Gainsbury et al. 2016; Houghton et al. 2019). Gainsbury (2015b) interviewed 19 gambling industry employees in Australia. Results suggested that from a gambling operator perspective, Twitter is the platform for immediate and urgent news concerning gambling. Gambling industry employees claimed that Twitter was a platform that they used to increase their player base by attracting new customers and retaining existing customers. Additionally, it was noted that there was a lack of responsible gambling messages when using the platform by gambling operators.

Gainsbury et al. (2016) examined the use of social media marketing by both online and land-based Australian gambling operators (n = 101). Twitter postings covered a range of topics, including information about the provider, sports news, special promotions and bets, and betting tips. Gambling operators presented gambling content alongside relevant news and events. Further analysis demonstrated numerous latent messages in the body of the social media posts which included (i) a lack of responsible gambling messages, (ii) increasing brand engagement, and (iii) aligning gambling with sport as a way of normalizing gambling.

Houghton et al. (2019) carried out the first quantitative content analysis of UK online gambling operators and gambling affiliate pages (gambling affiliates promote gambling websites, and in return they receive either commission or a percentage of profit). They identified nine content categories which included (i) betting assistance, (ii) direct advertising, (iii) sports content, (iv) customer engagement (tweets that would offer a poll to vote on or a pose a question, prompting a response from followers), (v) humor, (vi) an update of current bet status, (vii) safer gambling, (viii) promotional material and (ix) other. They found that compared to affiliate accounts, the Twitter accounts for the sports gambling operators posted more sports information content and posted more material containing humor. As with previous studies, the authors’ noted that there were few responsible gambling messages posted on Twitter.

Surveying an Australian sample (N = 964), Gainsbury et al. (2016) examined the engagement with gambling operators on social media (Facebook, Twitter, and YouTube) and the impact of social media marketing on their gambling behavior. They found that compared to non-risk gamblers, moderate risk gamblers and problem gamblers were more likely to have actively engaged with gambling operators on these platforms and more likely to report an increase in gambling due to these promotions. However, the data were collected via self-report, so whether these relationships exist in actuality remain to be confirmed.

There have been two notable reports that have investigated gambling operators and their use of social media. In a report commissioned by the Victorian Responsible Gambling Foundation (Australia), Thomas et al. (2015) researched different social media marketing strategies used by Australian gambling operators and noted that there was a high volume of promotions, which included strategies such as humor and engagement. However, such content may not have been thought of as promotional material by the consumers. Miller et al. (2016) produced a report commissioned the Responsible Gambling Trust (UK). The authors’ noted that gambling promotions, tips, and odds on Twitter were included as part of a more extensive discussion concerning sports (e.g., important matches, tactics, and player transfers). Additionally, 25% of tweets sent from gambling operators did not relate to gambling but included updates and humorous content from a range of different sports as well as commentary surrounding different sporting events and matches. The authors suggested that the integration of non-gambling related messages may be a contributing factor in the normalization of gambling due to it being an element of being a sports fan.

To date, no study has researched individual promotions and promotional strategies marketed by UK gambling operators. Furthermore, no research has examined how gambling operators utilize specific features on Twitter, including the use of hashtags within posted messages. Twitter data, in comparison to data from other social media platforms, such as Facebook, are more openly accessible for researchers to study. Because of this, there has been a recent increase in attention paid to advertising by gambling operators on Twitter (e.g. GambleAware 2019). Therefore, this platform was chosen over others to allow for the comparison of findings. The present study aimed to provide a snapshot content analysis of social media marketing on Twitter using data collected from the accounts of ten of the largest sports betting operators in the UK and to highlight any implications for regulation of gambling advertising in this digital market. More specifically, this study examined: (i) how gambling operators promote their products on Twitter; (ii) how Twitter features are used by gambling operators, such as the use of hashtags, (iii) how gambling operators interact with their followers, and (iv) implications that the findings may have on the regulation of sports betting advertising via Twitter.

## Method

Data for the study were analyzed through a content analysis of the Twitter sites of online gambling operators. Historic pages were accessed using the advanced search feature at twitter.com, allowing the authors to tailor the search results to specific date ranges. Before data collection commencement, a feasibility study was undertaken to assess the volume and nature of tweets produced.

The sample for the study was the largest bookmakers (by revenue) in the UK. These were: *Paddy Power, Bet365, William Hill, Coral, Ladbrokes, 888Sport, Betfair, Betfred* and *Unibet* (all of which have an online gambling product). Additionally, a decision was made to include *Sky Bet* in the sample due to its large number of Twitter followers at the time of the analysis (n = 365,133). *Paddy Power*, *William Hill, Coral, Ladbrokes and Betfred* also operate high street bookmakers. *Paddy Power, Bet365, Sky Bet, William Hill, Coral, Betfair, Betfred* and *Unibet* all offer other online gambling products (e.g., casino and/or poker), but these had separate Twitter pages and were only mentioned when it came to cross-posting from these sports dominated pages.

The present study focused on content posted on August 10–12, 2018, the start of the 2018–2019 English Premier League football [soccer] season (the top level of the English football league system). The English Premier League (EPL) was selected as the time point under study for several reasons. Firstly, the EPL is the most viewed sports league in the world, with the highest exposure of any sporting league. It has a potential television audience of 4.7 billion people (Dubber and Donaldson 2015) and it is broadcast in over 156 countries (Eurosport 2015). Secondly, the EPL has a strong brand for producing betting business (Forrest and Simmons 2003). Betting companies have become a major source of sponsorship for the EPL. For example, the EPL reached a soccer sponsorship high in the 2016/2017 season, with half of the football teams including a gambling operator (bookmaker or casino) on kit branding (Bunn et al. 2018). Additionally, football betting has the highest grossing gaming yield (£786 million in 2017) by sport for online sports betting and is considered to be the most popular betting activity (Gambling Commission 2018c). Therefore, it was anticipated that there would be a large volume of Twitter activity taking place at the start of the football season. Consequently, the findings may not generalize to other soccer leagues or sports.

The present study comprised a retrospective analysis of Twitter of all tweets sent by ten gambling companies between August 10 and August 12 (2018). Data collection took place between December 2018 and January 2019. The study used a directed and inductive approach to develop a coding template to analyse the Twitter posts. Additionally, a summative content analysis of social media promotions on Twitter was conducted (Hsieh and Shannon 2005). This approach involved the counting and comparisons of keywords, after which the underlying latent meanings were interpreted (Hsieh and Shannon 2005). Standard screen capture technology was used to take snapshots of Twitter profiles of the sample during the data collection periods. During this process, categories and variables used in previous research of a similar nature (i.e., Thomas et al. 2015) were adapted to suit the characteristics and aims of the present study. These were utilized when a coding sheet was created. For the present study, five critical areas of investigation were identified. Each theme had a series of different categories which were applied to each gambling operator, and coding frameworks were developed. The topics of inquiry included (i) user engagement, (ii) content of posting, (iii) responsible gambling messages, (iv) use of hashtags and (v) promotional content. These were compared between online-only bookmakers and land-based bookmakers which additionally operate online (to determine whether any differences were present).

More specifically:

- *User engagement* This documented the extent to which users engaged with content on Twitter. The following data were collated: number of followers; number of accounts following; total retweets; total favorites; total polls; total votes; and number of public tweet replies. Retweets can be viewed as a proxy for how many people are engaging with tweets. Having these messages retweeted by followers increases the exposure of the message. The frequencies for each of these categories were counted.
- *Content of posting* The nine content categories identified by Houghton et al. (2019) were applied to the present study, utilizing a deductive approach. These were *betting assistance* (promoting features that would help people with their betting, included betting tips provided by celebrities and match statistics to help people choose their bets); *customer engagement* (tweets that would pose a question, or offer a poll, prompting a response from followers); *direct advertising* (e.g., free bet offers and the provision of pre-match and in-play betting odds); *humor* (tweets that included a humorous tone when discussing things, such as sporting events); *promotional content* (sporting information that used relevant promotional hashtags); *responsible gambling* (responsible gambling messages offered by the operators); *sports content* (sports updates, sports news, sports reviews and commentary); *update of bet status* (promoting gambling wins from customers); and *other* (tweets that did not fit into the other content categories, for example, tweets discussing news stories and celebrity news).
- *Responsible gambling messages* This documented the extent to which responsible gambling messages were evident, either as a standalone message or embedded in other Tweets seemingly aimed at something else. For example, a sports betting offer may include responsible gambling details such as reference to the *begambleare.org* website (which promotes responsible gambling). This category took an inductive approach, where code categories were drawn directly from the data.
- *Use of hashtags* Hashtag use was measured by counting and categorizing individual hashtags which linked tweets to the wider Twitter audience. An inductive approach was taken to develop a coding scheme for this category.
- *Promotional content* The present study was also interested in examining the specific content of different promotions used by gambling operators, as has been done in previous research (i.e., Thomas et al. 2015). The present study also used an inductive approach to generate categories to be included in the coding framework. The following types identified were: pre-match odds; in-play odds; Twitter exclusive (where promotions are offered exclusively to their Twitter followers); requested odds; ‘cash out’ (a feature that allows a sports bettor to cash out their bet early for a profit if it is winning, or a smaller loss if it is losing); free bet offer (a bonus that is paid in the form of a free bet); mobile app promotion (a promotion that requires the mobile app to be downloaded in order for the person to qualify); loyalty card (part of a consumer incentive scheme which combines points collected in high street bookmakers and online); best odds guaranteed (where an early or fixed odds price is taken for a horse or greyhound race, but the starting price is greater, the bet is paid out at the bigger odds); bet builder (an automated version of manually requesting a sports bet); requested odds (a feature that allows the bettor to request odds); and a cash prize competition.

In terms of coding used for promotion content, in-play betting allows bettors to place a bet on an event once it has started. To distinguish when these odds were promoted via tweets, they were identified as those offered during a sporting event by either stating that the odds are in-play in the body of tweet (e.g. *“In*-*play. Manchester United V Leicester. Anytime Goalscorer*- *Juan Mata, 9/2”(William Hill,* 10th August, 2018), a hashtag referring to in-play betting (e.g., “HT: Wolves 1-1-Everton. Diogo Jota is 7/1 to score next #InPlaywithRay”), or an in-play market is offered (e.g., next goal scorer, next team to score, who will win the next corner?). Pre-match betting refers to bets placed prior to an event starting. These are identified by stating they are pre-match odds or showing odds for an event which has not started yet (which can be identified by the time the tweet is posted vs. the starting time of the event). Requested odds, a feature which allows users to request odds for a particular selection on a chosen event, was identified by hashtags that were relating that specific gambling company (*William Hill*- #yourodds, *Ladbrokes*- #getaprice, *Coral*- #yourcall, *Betfred*- #pickyourpunt and *Paddy Power*- #whatoddspaddy) followed by a link to the betting odds.

An odds request on Twitter is a reply to a request for individual odds that has been requested by a customer, and upon replying, the new market is shared with other customers on the gambling operator’s webpage. Additionally, links to a market of requested odds were also coded as requested odds, for example: “*The Premier League is back tonight. Latest #WhatOddsPaddy specials for tonight’s Man Utd v Leicester game: pdy.pr/LZnEZo*” (*Paddy Power,* 10 August, 2018). Rejected odds requests were also coded for in this category. For example, if the gambling operator replied to the customer that they would not be able to offer the requested odds. T-tests was carried out to assess whether there was a statistically significant difference between the Twitter postings of online-only bookmakers and land-based bookmakers which additionally operate online. Chi-square tests were then carried out to determine whether there was a significant association between the type of bookmaker and the promotional strategies employed.

## Results

### Sample Characteristics

The ten operators included in the sample were: *Paddy Power, Bet365, Sky Bet, William Hill, Coral, Ladbrokes, 888Sport, Betfair, Betfred* and *Unibet* (all of which have an online gambling product). *Paddy Power, Bet365, Sky Bet, William Hill, Coral, Betfair, Betfred* and *Unibet* all offer other online gambling products (e.g. casino and/or poker), but these had separate Twitter pages and were only mentioned when it came to cross-posting from these sports dominated pages. *William Hill, Ladbrokes, Coral, Paddy Power* and *Betfred* also have high street land-based bookmaking shops. A total of 2,527,509 accounts followed sports betting operators on Twitter. It is possible that individual Twitter users followed more than one account (e.g., a user can follow more than one bookmaker). The number of followers of sports betting operators on Twitter ranged from to 1540 (*Unibet*) to 643,499 (*Paddy Power*).

Many independent samples t-tests were conducted using Bonferroni adjusted alpha levels of .0015 to examine whether there was a statistically significant difference between online bookmakers and land-based bookmakers that also operated online (Table 1). Results showed no significant differences between the two types of bookmaker.

**Table 1:** Comparison of Twitter content between ‘land-based and online bookmaker’ vs. ‘online-only bookmaker’

	Land-based and online bookmaker (n = 5)	Online- only bookmaker (n = 5)	Sig.^a^		Land-based and online bookmaker (n = 5)	Online-only bookmaker (n = 5)	Sig.^a^
	Mean (SD)	Mean (SD)			Mean (SD)	Mean (SD)	
*Different types of content posted*							
Betting assistance	5.80 (5.63)	2.80 (2.78)	.32	Customer engagement	333.40 (428.49)	42.20 (72.25)	.17
Direct advertising	78.20 (31.82)	23.00 (22.75)	.01	Humor	36.20 (22.54)	19.60 (19.93)	.25
Other	3.00 (1.87)	.40 (.55)	.02	Promotional	4.20 (2.59)	1.20 (1.30)	.49
Responsible gambling	1.80 (1.79)	3.00 (3.00)	− .77	Sports content	135.40 (72.90)	70.00 (67.54)	.18
Update of bet status	.80 (110)	1.10 (1.60)	.55				
*Responsible gambling Twitter postings*							
RG post	1.60 (1.52)	3.00 (3.00)	.38	RG message embedded	46.80 (27.45)	4.00 (5.87)	.01
*Consumer engagement*							
Total retweets	3177.80 (4849.10)	2352.20 (3755.24)	.77	Total favorites	1654.20 (28,915.17)	9666.40 (14,700.27)	.47
Number of polls	3.60 (1.67)	4.20 (4.60)	− .27				
*Promotional strategies*							
Enhanced odds	23.00 (19.43)	3.00 (2.83)	.52	In-play odds	17.40 (10.31)	8.20 (14.53)	.28
Pre-match odds	19.20 (9.94)	3.20 (3.96)	.01	Twitter exclusive	2.60 (2.61)	2.20 (3.49)	.84
Requested odds	192.60 (297.20)	0 (0)	.19	Tipping	2.20 (2.68)	.80 (1.30)	.33
Cashout	0 (0)	.40 (.89)	.35	Free bet offer	3.40 (3.29)	1.40 (1.34)	.24
App promo	.60 (1.34)	0(0)	.35	Loyalty card	.80 (1.79)	0 (0)	.35
Best odds guaranteed	1.00 (1.73)	.20 (.45)	.80	Facebook promotion	2.40 (2.51)	0 (0)	.07
Bet builder	1.20 (2.68)	1.00 (1.41)	.89	Cash competition	0 (0)	.10 (.89)	.35
*Hashtag Twitter posts*							
Football match	37.00 (48.98)	16.20 (13.57)	.92	National football league	10.20 (19.51)	6.80 (8.44)	.73
Football team name	32.60 (30.29)	15.80 (17.43)	.31	Boost promotion	8.80 (15.87)	2.80 (4.38)	.81
Requested odds	198.60 (310.33)	0 (0)	.19				

*Note*: All t-tests were tested upon 8 degrees of freedom

^a^Adjustments for multiple comparisons: Bonferonni correction

Chi-squared tests were carried out to assess whether the proportion of posts, which included different promotional strategies, content categories, hashtag use. and responsible gambling messages differed significantly between online bookmakers and land-based bookmakers which also operate online. Results showed no significant differences concerning the frequency of posts between the two bookmaker groups.

### User Engagement

All operators provided multiple tweets per day during the three-day period, ranging from 33 tweets per day (*Betfair*) to 398 tweets per day (*William Hill*) as shown in Table 2. The tweets contained multiple forms of content, with over one-third of all tweets featuring a picture (n = 1294), and around 3% of tweets included a video (n = 128) or graphics interchange format [GIF] (n = 120). A GIF within this context typically includes moving images with no sound.

**Table 2:** User engagement by sports betting operators on Twitter [Part 1] (August 10–12, 2018)

	Paddy Power (n = 274)	Bet365 (n = 490)	Sky Bet (n = 88)	William Hill (n = 1194)	Coral (n = 469)	Ladbrokes (n = 648)	888Sport (n = 112)	Betfair (n = 33)	Betfred (n = 402)	Unibet (n = 65)	Total (n = 3375)
Followers	643,499	381,700	365,133	208,433	333,334	197,370	1540	158,298	106,419	131,783	2,527,509
Following	2550	365	268	35	12,300	1299	1718	1132	889	1016	21,572
Total tweets	221,397	341,698	612,322	415,767	288,151	189,309	25,116	117,540	229,764	81,180	2,522,244
Tweets (August 10–12)	274	490	88	1194	469	648	112	33	402	65	3775
Average tweets per day	91.3	163.3	29.3	398	156.3	216	37.3	11	134	21.7	1258.20
Images	75	255	53	75	210	264	81	28	223	30	1294
Video	0	15	8	0	27	36	2	2	33	5	128
GIF	13	3	12	4	22	25	14	0	17	10	120

The user engagement with tweets varied between operators (see Table 3). Tweets were retweeted a total of 27,650 times. Tweets from *Paddy Power* were the most shared (total of 11,560 which is an average of 42.2 retweets per tweet, while the average across all the operators was 8.2 retweets per tweet). A total of 131,043 posts were ‘favorited’ in total, with the highest number being favorited on the *Paddy Power* account (n = 67,861). Across all brands, 149,721 votes were made in various polls, an average of 3839 votes per poll. *Bet365* had the highest average number of votes per poll (n = 9390).

**Table 3:** User engagement by sports betting operators on Twitter [Part 2] (August 10–12, 2018)

	Paddy Power (n = 274)	Bet365 (n = 490)	Sky Bet (n = 88)	William Hill (n = 1194)	Coral (n = 469)	Ladbrokes (n = 648)	888Sport (n = 112)	Betfair (n = 33)	Betfred (n = 402)	Unibet (n = 65)	Total (n = 3375)
Total # retweets	11,560	8851	2345	156	3204	762	473	0	207	92	27,650
Total favorites	67,861	34,678	11,184	695	9486	4079	1909	15	590	546	131,043
Number of polls	1	11	6	3	4	5	4	0	5	0	39
Total votes	6064	103,297	12,805	3448	7598	14,990	666	0	853	0	149,721

### Responsible Gambling Message

Only .68% of the total tweets contained a message solely focused on responsible gambling. Only six operators (*Bet365, Sky Bet, William Hill, Coral, Ladbrokes* and *Betfair*) tweeted a responsible gambling message. These messages contained information or advice relating to responsible gambling, for example: *“Don’t chase your losses*—*stay in control. Gamble responsibly”* (*Bet365,* August 12, 2018). Alongside this was often a hyperlink which said: *“Click here for more information on Responsible Gambling”*, which would then direct individuals who clicked on it to the gambling operator’s responsible gambling support page or an independent gambling help organization. These messages were not embedded with any other content and were standalone messages. Responsible gambling information embedded within other promotions (e.g., free bet offers), was provided by seven of the ten gambling operators (*Paddy Power, Bet365, Sky Bet, William Hill, Coral, Ladbrokes* and *Betfred*), accounting for 7.5% of tweets (Table 4). Examples of responsible gambling information included a link to *begambleaware.org* (an independent charity funded by donations from British gambling operators who fund problem gambling treatment, education, and research in the UK) or a message from The Senet Group (an independent body that promotes responsible gambling practices; e.g. *“When the fun stops, STOP”*).

**Table 4:** Responsible gambling messages by different sports betting operators on Twitter (August 10–12, 2018)

	Paddy Power (n = 274)	Bet365 (n = 490)	SkyBet (n = 88)	William Hill (n = 1194)	Coral (n = 469)	Ladbrokes (n = 648)	888Sport (n = 112)	Betfair (n = 33)	BetFred (n = 402)	Unibet (n = 65)	Total (n = 3375)
RG stand-alone	0	6	6	3	3	2	0	3	0	0	23
RG embedded	2	13	7	39	64	61	0	0	68	0	254

### Content of Posting

Sports content was the most commonly posted (n = 1027, 26.93%), with *Ladbrokes* posting the higher amount (n = 207) (Table 5). For example: *“Man Utd XI: De Gea, Darmian, Bailly, Lindelof, Shaw, Fred, Pogba, Andreas, Mata, Sanchez, Rashford”* (Bet365, August 10, 2018). Promotional content was the second most comment content type posted (n = 990; 25.96%). A popular social media strategy was to post promotional content using specific hashtags, for example, to promote specific bet requests. Customer engagement was the third most common content type posted (n = 915; 24%). For example, “*Will Harry Maguire score against Manchester United?”* (*Bet365,* 10th August, 2018) is an example of a poll in a tweet by *Bet365*, whereby users may click their preferred options (in this case, voting on whether or not they predict that a soccer play will score in the specified game). The results are immediately displayed after the vote and they can only vote in a poll once.

**Table 5:** The number of tweets made within each content category by bookmaker (August 10–12, 2018)

	Paddy Power (n = 274)	Bet365 (n = 490)	Sky Bet (n = 88)	William Hill (n = 1194)	Coral (n = 469)	Ladbrokes (n = 648)	888Sport (n = 112)	Betfair (n = 33)	BetFred (n = 402)	Unibet (n = 65)	Total (n = 3375)
Betting assistance	15	4	2	0	6	3	0	7	5	1	43
Customer engagement	51	171	12	373	89	112	19	5	79	4	915
Direct advertising	54	63	11	49	70	91	12	9	127	20	506
Humour	68	54	15	6	44	33	13	2	30	14	279
Other	3	0	0	1	2	6	1	0	3	1	17
Promotional content	8	1	3	714	64	168	0	0	30	2	990
Responsible gambling	0	6	6	4	3	2	0	3	0	0	24
Sports content	96	186	42	32	192	207	69	16	150	37	1027
Update of bet status	0	2	6	0	0	2	0	0	2	0	12

### The Use of Hashtags and Links with Sport

The following section presents the findings related to the hashtags used within each of the Twitter pages (Table 6).

**Table 6:** Hashtags used by different sports betting operators on Twitter (August 10–12, 2018)

Type of hashtag	Paddy Power (n = 274)	Bet365 (n = 490)	Sky Bet (n = 88)	William Hill (n = 1194)	Coral (n = 469)	Ladbrokes (n = 648)	888Sport (n = 112)	Betfair (n = 33)	Betfred (n = 402)	Unibet (n = 65)	Total (1905)
*Relating to a specific match or game*											
Basketball	0	0	0	0	0	0	1	0	0	0	1
Darts	0	0	0	0	0	0	0	0	1	0	1
Snooker	0	0	0	0	0	0	0	0	2	0	2
UFC	0	0	0	0	0	0	0	1	0	0	1
Cricket	0	2	1	1	3	8	3	0	3	2	23
Horse racing meeting	4	0	0	0	0	2	0	0	0	0	6
Boxing	0	0	0	0	0	0	0	0	1	0	1
Football	16	4		10	0	38	28	4	121	33	263
*League/competition*											
International football	0	0		0	0	0	0	0	0	0	1
Golf competition	0	3	5	8	15	22	1	1	3	2	60
Rugby League	0	0	0	0	0	0	0	0	26	0	26
National football league	0	0	10	3	0	3	0	4	45	20	85
Tennis competition	0	0	0	0	0	3	0	0	2	2	7
Deadline day	0	0	1	0	0	0	0	0	1	0	2
*Individual*											
Football manager	0	0	0	0	0	0	0	0	1	0	1
Football player	0	0	0	0	0	0	0	0	2	0	2
Football team name	2	5	25	8	25	57	7	0	71	42	242
Golf player	0	0	0	3	0	0	0	0	0	0	3
Cricket team	0	0	0	0	0	0	0	0	0	1	1
*Promotional*											
Boost promotion	0	0	10	3	0	4	4	0	37	0	58
Responsible gambling	0	0	5	3	3	1	0	3	0	0	15
Requested odds	6	0	0	743	56	163	0	0	25	0	993
Other promo	5	0	3	3	6	20	0	0	10	0	47
In-play	0	26	0	0	0	0	0	0	3	0	29
Best odds Guaranteed	0	0	0	0	0	0	0	0	1	0	1
*Other*	2	4	0	4	6	5	2	0	10	1	34

In total, 1870 hashtags were used and there could be more than one hashtag per Tweet. Over half of the hashtags came from William Hill (n = 743, 52.13%) and were related to odds requested by customers as show in Table 5. In total, five of the ten gambling operators tweeted prices for selections that had been requested. These were often accompanied by individual tweets from gambling operators. *William Hill:* Your odds (#yourodds, n = 743, 52.13%), *Ladbrokes:* Get a price (#getaprice, n = 163, 8.56%), *Coral:* Your call (#yourcall, n = 56, 2.94%), *Betfred*: Pick your punt (#pickyourpunt, n = 25, 1.31%), and *Paddy Power:* What odds Paddy? (#whatoddspaddy, n = 6, .35%).

All of the gambling operators in the present study linked their tweets to sporting matches using game-based hashtags. Operators used these hashtags to embed the tweet into existing Twitter feeds about a specific game. The most commonly used type of hashtag was linked to a specific football match (n = 263; e.g., #DCFCVLUFC [Derby County Football Club vs. Leeds United Football Club]. Just under half of these tweets (n = 121) were tweeted by *Betfred.* The second most popular type of hashtag linked the tweet to a specific football team or teams (n = 242, 13.81%; e.g. #LUFC [Leeds United Football Club]. A total of 594 hashtags (64.08%) related to football in some way (player, competition, team, manager). A national football league was the most commonly mentioned type of competition (n = 85), followed by golf (n = 60), and then rugby league (n = 26).

### Promotional Content

Twitter-based promotions were classified into 13 categories: pre-match odds, in-play odds, Twitter exclusive, tipping, requested odds, cash out, free bet offer, mobile app promotion, loyalty card, best odds guaranteed, bet builder, and a cash prize competition (Table 7).

**Table 7:** Promotional content by different sports betting operators on Twitter (August 10–12, 2018)

	Paddy Power (n = 274)	Bet365 (n = 490)	Sky Bet (n = 88)	William Hill (n = 1194)	Coral (n = 469)	Ladbrokes (n = 648)	888Sport (n = 112)	Betfair (n = 33)	Betfred (n = 402)	Unibet (n = 65)	Total (n = 3375)
Odds boost/enhanced odds	0	0	6	13	19	32	2	6	51	1	130
Pre-match odds	21	8	0	5	14	26	1	0	30	7	112
In-play odds	16	34	0	8	13	15	4	0	35	3	128
Twitter exclusive	0	8	3	3	4	6	0	0	0	0	24
Requested odds	5	0	0	713	56	163	0	0	26	0	963
Tipping	0	3	1	0	6	1	0	0	4	0	15
Cash out	0	0	0	0	0	0	0	2	0	0	2
Free bet offer	6	3	2	0	4	7	0	0	0	2	24
App promo	0	0	0	0	0	3	0	0	0	0	3
Loyalty card (the Grid)	0	0	0	0	0	4	0	0	0	0	4
Best odds guaranteed	0	1	0	0	0	4	0	0	1	0	6
Facebook promo	3	0	0	0	6	3	0	0	0	0	12
Bet builder	0	3	0	0	0	0	0	2	6	0	11
Cash competition	0	0	0	0	0	0	0	0	0	2	2

Supplying specifically requested odds was the most popular form of promotion (n = 963; 67.06%). Betting customers have the option to ask for a specific price for a bet. These odds are then supplied on Twitter by the gambling operator who use a relevant hashtag. The odds are then made accessible on site for the requested (or any other) person who wants to place a bet on that market to access.

The second most commonly tweeted promotion was the provision of enhanced or boosted odds (n = 130; 9.05%). These were most commonly used by *Betfred* (n = 51), *Ladbrokes* (n = 32) and *Coral* (n = 19). Some tweets promoted increased odds in order to encourage people to bet, for example, a tweet from @Coral which says *“HALF*-*TIME SMART BOOST. Ruben Neves To Score a Brace*-*8/1 (Was 6/1)* (August 11, 2018).

The third most common type of promotion on Twitter from the selected operators was the provision of in-play sporting odds information (n = 128, 8.91%). For example, some tweets would show the current in-play betting odds for a specific match and there would be a hyperlink to the betting odds, such as *“Underway! Salah is 9/4 to score first in play! Bet here ≫ fal.cn/VSdb”* (*Betfred*, August 12, 2018). This was most commonly used by *Betfred* (n = 35), *Bet365* (n = 34) and *Paddy Power* (n = 16). Another popular type of odds promotion was encouraging individuals to bet before a match by providing odds before a game started or a league was underway (n = 121, 8.42%). This was most commonly used by *Betfred* (n = 30), *Ladbrokes* (n = 26) and *Paddy Power* (n = 21).

## Discussion

The present study sought to provide a snapshot content analysis of social media marketing on Twitter among the largest online sports betting operators in the UK. Results show that there was a large number of tweets being posted by gambling operators on Twitter, with one operator (i.e., *William Hill*) averaging over 390 tweets per day. Multiple hashtags were used which linked the tweets with popular sporting events and emphasized betting promotions. Analysis demonstrated that there was a wide variety of different promotional strategies employed, and that the number of responsible gambling messages were few.

The present study found that the number of responsible gambling messages—either standalone or embedded within the body of a Tweet—concerning something else (typically promotions) were few (8.51%). These findings support previous research which has reported that responsible gambling messages included within online gambling adverts are sparse (Hing et al. 2015) and not prevalent on Twitter profiles and postings for sports betting operators (Gainsbury et al. 2016; Houghton et al. 2019).

The findings demonstrated that multiple different promotions were employed and advertised by online sports betting operators. Newall et al. (2019) suggested that gambling marketing usually fits into one of three categories: (i) brand awareness, (ii) financial incentives, and (iii) odds advertising. Within the present study, the promotion of requested odds, which falls into the category of ‘odds advertising’, was found to be the most prevalent form of promotion. One theory as to why customers request individual odds is that they could be classified as a “market maven” (Feick and Price 1987), an individual who conscientiously absorbs and acquires information about numerous products on a continuous basis and due to this knowledge believe they have an influence over other customers (Williams and Slama 1995). How this may apply to the requesting of sports betting odds is that gambling operators can communicate these odds directly to the influential consumer, and as a result this information is spread with others on their Twitter network, who trust that specific individual’s knowledge concerning gambling. This allows the sports betting operator to spread a marketing message at low financial cost. Conversely, offering sports bettors the option to choose further betting options could lead to an overestimation in their ability to predict a sporting outcome, and result in cognitive distortions (Griffiths 1994) such as the illusion of control (Langer 1975). The online sports betting operator may benefit from responding to such customer tweet requests. For example, in the airline industry, Huang (2015) found benefits from responding to a Tweet included higher satisfaction than other customer service channels, the individual being more likely to recommend the operator to a friend or family, and potential for higher revenue if tweeted back quickly.

Another commonly used type of gambling marketing utilized in this study was the advertising of odds, in particular, in-play sports betting odds. Previous research has found that in-play sports betting has the potential to be more dangerous than other mechanisms of sports betting and encourages impulse sports betting (Killick and Griffiths 2018). Gambling operators use numerous types of promotion. For example, Hing et al. (2015) identified 15 different types of betting promotion (e.g., mobile betting bonuses, refund/stakeback offers, happy hours, and multi-bet offers). Similarly, the present study identified promotions such as free bet offers, including ‘money back’ offers (which returned the stake as a free bet is the bet was a losing bet), ‘cash out’ (a feature that allows somebody to settle their bet early, for a profit if it is winning, loss if the best is losing) and matched bet offers (which match/partially match the stake or deposit with bonus bets). These promotions may contribute to gamblers thinking they are less likely to lose and that they are receiving greater value for money, therefore, diminishing concerns that sports bettors may lose their money and contribute to a reduction in perceived risk that is usually associated with gambling. (Thomas et al. 2015).

The findings compliment those of Houghton et al. (2019) who found that the most common type of Twitter posting for online gambling operators was categorized as sports content (39.59%), which included sports news, interviews, and match commentary. It is important to note that there is a crossover between the gambling operators used within this study and by Houghton et al. (2019). However, the present study expanded on the findings by increasing the number of British gambling operators studied from five to ten.

Online sports betting operators often engage and provide content to users at the same time as live sporting moments within the sporting event. The hashtag is often used to draw attention to the sporting event. They can also help in the organization and promotion of tweets, and also designate that a particular tweet is about the same topic as all of the additional tweets using the same hashtag (Zarrella 2010). A large number of tweets in the present study had game-related hashtags which directly linked the tweets to specific sporting games, which support the findings of Thomas et al. (2015). These tweets were also targeted at sports fans who might be reading commentary about the game on social media (Thomas et al. 2015). Thomas et al. 2015 suggested that doing this may attract people to bet when they had not originally planned to gamble. Therefore, it might be worth systematically investigating the timing and content of promotional messages (Thomas et al. 2015). The present study found that the three most commonly used hashtags linked to tweets were those containing (i) requested betting odds; (ii) a specific game, and (iii) a specific team.

The gambling operator connects potential betting opportunities to the sporting event by including a sport-themed hashtag as part of that conversational topic. A hashtag linking the tweet to a national football league was the most commonly mentioned type of competition (n = 85, 4.46%) which was unsurprising given that the data were collected during the start of the English Premier League football [soccer] season. Previous research found that tweets are more likely to be retweeted if they contain hashtags (Suh et al. 2010). Here, the sports betting operator would be spreading their message to a broader audience and increasing the interactivity of the tweet. Furthermore, a hashtag links the tweet to a specific topic, and therefore with positive associations that are connected to those topics. Research has found that the liking of a stimulus, (e.g., a brand) can increase when a stimulus has been paired with other positive stimuli (De Houwer et al. 2001). The inclusion of such hashtags allows the potential for that those under the age of 18 years who are searching Twitter for content on a topic not related to gambling (e.g., a football event), may be exposed to gambling promotions and this may encourage some of them to visit gambling websites.

Previous research by Miller et al. (2016) identified six communities of gambling enthusiasts that formed online. It was reported that the central cluster, ‘the main bookmakers cluster’, was responsible for over 80% of all retweets to (or mentioning a) gambling account. Three-quarters of this cluster group (75%) were male. Identifying these clusters allows for further understanding of how bettors use Twitter. Although this has not been addressed in the present study, future research could investigate who was responsible for retweets (i.e., individuals or other gambling operators).

The present study also supports the idea of the gambling industry being embedded within sport, which has also been termed the ‘gamblification of sport’ (Lopez-Gonzalez and Griffiths 2017). Similarly, alcohol marketing on social media often inserts the word ‘alcohol’ into the conversations and daily routines of consumers, and as a result normalises alcohol (Nicholls 2012). The normalisation of products is a tactic that appears to be employed within gambling marketing. This idea is supported by Gainsbury et al. (2015a) who asserted that online gambling operators produce online gambling content alongside sports news and events, and arguably irrelevant content, to normalize gambling in a broader social context.

To date, only one study (i.e., Houghton et al. 2019) has provided an overview of how social media, specifically Twitter, is being utilized by UK sports betting operators, and the content of the messages that are being conveyed to the online Twitter community. The present study builds upon prior work assessing how online sports betting operators use Twitter utilizing content analysis (Gainsbury et al. 2015a; Houghton et al. 2019). A key strength of the present research is that data were analyzed across multiple categories, some of which have not been previously explored in the context of the UK sports betting marketing, including Twitter promotional strategies and hashtag usage. While some notable findings were made, it is not possible to make definitive conclusions about whether individuals interacting and/or following online sports betting Twitter accounts impacts on their gambling behavior online and/or offline.

The issue of whether the different promotions posted on Twitter classify as advertising is debatable. If a company promotes ordinary tweets (that is when tweets are paid for by advertisers in order to initiate engagement from existing customers or to reach a larger audience), these will be identified as they will be labelled as “promoted” and are advertising. Similarly, posts such as the direct advertising of betting odds can clearly be identified as advertising. On the other hand, other types of content, such as ‘humorous’ posts, and whether these classify as advertising is debatable. Many adverts are designed to create a sense of awareness for a brand, rather than directly influencing a betting decision immediately. For example, *Paddy Power* has created a reputation for engaging people with humorous content, appealing to its audience. As a result, this influences the number of ‘likes’ and retweets, increasing the brand’s Twitter presence. Therefore, if advertising is the process in which an organisation encourages people to engage in betting products or services, including the drawing of attention to the product and building brand awareness, it could be argued that Twitter posts in the present study can be classified as advertising

### Limitations of the Present Study

The first author developed an initial coding scheme using 50 tweets from each of the ten accounts and applied this coding scheme to the remaining data. The second author reviewed the tweets to make sure that there was agreement. One methodological weakness was that inter-rater reliability was not calculated for inductive analysis which may affect the validity of the findings. Additionally, Twitter was the only social media platform studied, limiting the generalizability of findings to other social media platforms. The data collection for the present study was cenetered upon one main sporting competition, therefore, the results are not generalizable to other sports or necessarily the same sport in other countries. A limitation of Twitter data in the present study is that they do not offer information on the effects of tweets on subsequent behavior. The extent to which Twitter users were exposed to sports betting marketing posts is not known. The traditional self-report survey that assesses excessive gambling behavior can be time-consuming for researchers and responders, susceptible to biases, and may have a low response rate. Social media arguably offers a real-time, large-scale examination of gambling behaviors and attitudes, with very few restraints. However, because the data were collected retrospectively, it is important to note that tweets remaining may not represent the initial number of tweets posted (e.g., tweets may have been deleted prior to data collection). Whilst the present research provides a snapshot of how gambling products are being marketed on Twitter, this particular study does not shed light on whether these advertisements actually impact gambling behavior. However, it has been established that advertising is one of a number of environmental factors that may affect gambling behavior concurrently (Griffiths and Parke 2003; Parke et al. 2015), which makes it hard to try and ascertain the exact role of social media advertising when it comes to gambling-related harm.

## Conclusions and Future Directions

Based on the findings of the present study, examining the content of posts on Twitter may provide valuable insights into how information about sports betting products are marketed via social media. The results here complement previous research that has shown that numerous marketing strategies are employed, and that responsible gambling messages are infrequent. Sporting hashtags were used by gambling operators to tie in social media posts with key sporting events. Therefore, it will be essential for researchers to examine the content of sports betting advertising tweets, such as frequency of tweeting and the content of tweets. Twitter serves as a platform where gambling operators can market their product in a normalized and positive way. Future research could examine the gambling consumers and their response to the Twitter postings in addition to those of the gambling operators.

New 2018 British regulations require that all broadcasted gambling adverts feature a responsible gambling message or reference to www.begambleaware.org throughout the advertisement. It is further suggested that all gambling content and communication should include their website information (including that on social media), so individuals know where to access information, support, and advice. There needs to be a review of regulatory policy for advertising gambling products via social media, possibly something to a similar affect. The development of effective policy will need to consider restriction on the availability of gambling advertisements on this social media platform that is likely accessed by children. One method, as suggested in a report by GambleAware (2019), is to introduce age screening tools before individuals can follow accounts that relate to or promote gambling. Additionally, betting companies and advertisers could better utilize adtech in order to remove online betting profiles that have a high chance of being shown to a child (GambleAware 2019). Future research could examine particular creative strategies used by social media operators, for example, the use of humor, and how the use of these strategies influence the intentions and attitudes towards gambling from children and other vulnerable and susceptible groups. The present research contributes to the awareness of content posted on social media by gambling operators and provides data for policymakers and decision-makers with the aim of adopting regulatory frameworks which reduce gambling harm.
